# Cancer and Thrombosis—New Insights

**DOI:** 10.5041/RMMJ.10349

**Published:** 2018-10-04

**Authors:** Yona Nadir, Benjamin Brenner

**Affiliations:** Thrombosis and Hemostasis Unit, Department of Hematology, Rambam Health Care Campus, Haifa, Israel

**Keywords:** Cancer, coagulation, heparanase, heparins, TFPI-2 peptides

## Abstract

Cancer patients have a pro-thrombotic state attributed to the ability of cancer cells to activate the coagulation system and interact with hemostatic cells, thus tilting the balance between pro- and anticoagulants. Mechanisms underlying the coagulation system activation involve tumor cells, endothelial cells, platelets, and white blood cells. Anti-cancer therapies, including anti-angiogenic drugs, significantly increase the risk of thrombosis during treatment. Along with the role of coagulation proteins in the hemostatic system, these proteins also serve as growth factors to the tumor. Heparanase is a pro-angiogenic and pro-metastatic protein. Our previous studies have demonstrated that it enhances tissue factor (TF) activity and is present at high levels in tumor cells and patients’ blood. Strategies to attenuate heparanase effects by heparin mimetics or peptides interrupting the TF–heparanase interaction are good candidates to attenuate tumor growth and thrombotic manifestations.

## INTRODUCTION

Activation of the coagulation system in cancer patients is very common. Activated coagulation proteins not only contribute to clinical thrombotic manifestations but also serve as growth factors to tumor and endothelial cells. In addition, proteins released by the tumor, such as cytokines, can activate the coagulation system, imposing a positive feedback between cancer and coagulation. Knowledge is accumulating on the mechanisms and the effects of the hemostatic system on tumor development, and attempts to attenuate these effects are ongoing.

## EPIDEMIOLOGY OF CANCER AND THROMBOSIS

Thrombotic manifestations are a common cause of morbidity and mortality in cancer. Patients with various types of malignancies frequently demonstrate aberrations in all the elements of Virchow’s triad, including endothelial damage*,* blood stasis, and changes in the blood coagulation factors and cells*,* which may result in a prothrombotic or hypercoagulable condition. Clinical presentations range from localized venous or arterial occlusion to systemic syndromes, such as disseminated intravascular coagulation (DIC) and thrombotic thrombocytopenic purpura (TTP). Pulmonary embolism is known to be a major cause of death in cancer patients.

It is well established that about one-quarter of venous thromboembolism (VTE) patients suffer from cancer.^1^ Among 1,200 patients with cancer-associated VTE diagnosed at the Rambam Medical Center between 2006 and 2017, two-thirds were ambulatory. The risk of VTE depends on the cancer type, site, and stage, with the highest incidence observed in patients with pancreatic, gastric, brain, and ovary tumors; in patients with lymphomas; and in those with metastatic disease.^2^ According to the Danish National Registry, the diagnosis of cancer-associated VTE is a predictor of poor prognosis.^3^ Patient-related factors, such as co-morbidities and therapy-related factors (surgery and chemo-hormonal, radio-hormonal, and anti-angiogenic therapies), contribute to the increased thrombotic risk.

Several studies demonstrated an increased risk of subsequent arterial ischemic events in patients with VTE.^4^,^5^ Notably, recent studies reported an increased risk of arterial ischemic events in cancer patients.^6^,^7^ Vascular complications in patients with VTE and active cancer also include arterial ischemic events.^8^ The probability of vascular events is likely to increase with longevity associated with the introduction of novel treatment modalities^9^ and improved patient survival rates. One should bear in mind bleeding complications, which are more common in cancer patients receiving anticoagulant therapies.^10^

## MECHANISMS INVOLVED IN CANCER AND THROMBOSIS

Various factors contribute to the activation of the hemostatic system.^11^ Circulating tumor cells over-expressing tissue factor (TF) may activate the hemostatic system on entering the blood circulation as whole cells or as microparticles exposing TF on their surface. Microparticles may originate from tumor cells, platelets, white blood cells (WBCs), and endothelial cells. Mucins are large oligosaccharides that are usually secreted by epithelial cells of organs with a lumen, such as the gastrointestinal tract. Their primary role is to lubricate and prevent bacteria from entering the blood circulation. Once epithelial cells turn into carcinoma cells, their mucins are also secreted into the circulation. In the blood, these large sugar molecules interfere in the WBC interaction with the endothelium and activate the platelets. These activated platelets release the heparanase protein that further enhances TF activity. Cytokines, such as tumor necrosis factor (TNF), interleukin (IL)-1, and IL-6, are secreted from tumor cells and induce exposure of TF and release of microparticles in endothelial cells. Acquired activated protein C resistance due to elevated factor VIII found in cancer patients further contributes to the coagulation system activation.^11^–^13^

**Figure 1:**
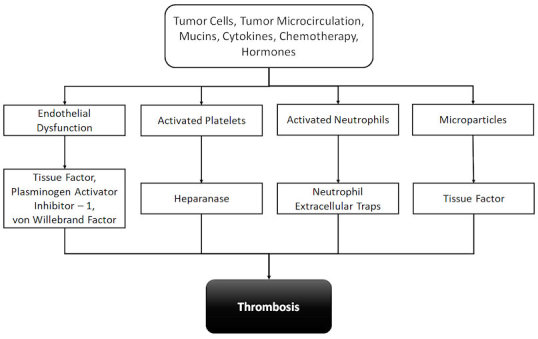
Mechanisms of Thrombosis in Cancer Various components released from tumor cells induce coagulation system activation either directly through TF or indirectly through the effect on endothelial cells, platelets, and neutrophils.

Recently, the involvement of neutrophil extracellular traps (NETs) in cancer thrombosis was described.^14^,^15^ These NETs, composed of chromatin fibers, were shown to form a scaffold to platelets and red blood cells, facilitating the thrombus formation. The pathologic microcirculation in the tumor, depicted by abnormal morphology and flow, further contributes to the hemostatic system activation.^16^ The vessels in the tumor were demonstrated to be dilated with excess branching, resulting in blood stasis. Due to the low flow, growth factors, and cytokines released from the tumor cells, endothelial cells expose TF on their surface and release von Willebrand factor (vWF) and plasminogen activator inhibitor (PAI-1). At therapy initiation, there is a significant increase in the risk of thrombosis attributed to the effect of chemotherapy, antiangiogenic drugs, and hormones.^17^ Drug-induced thrombosis involves a variety of mechanisms, including enhancement of procoagulant activity, reduction in anticoagulant synthesis, simulation of platelet aggregation, and endothelial damage.^17^ A summary of these mechanisms is presented in Figure 1.

## HEPARANASE PROTEIN INHIBITION AND HEPARINS AS ANTI-CANCER TREATMENTS

### Heparins as an Anti-tumor Therapy

The commonly used heparins include the unfractionated heparin (UFH) sourced from animal tissues, and biochemically derived low-molecular-weight heparins (LMWHs). The primary effect of heparins on the coagulation system is related to the inhibition of thrombin and factor Xa action. The mechanisms of heparin-mediated anti-tumor effects are more complex and involve growth factor release from the endothelial cell surface, obstruction of P- and L-selectin-associated cell adhesion, heparanase protein inhibition, and reduction in the coagulation system activity. The main potential shortcoming of heparins is their non-specific interactions with proteins, such as growth factors and selectins. For instance, tissue factor pathway inhibitor (TFPI), which is both an anti-angiogenic and anti-metastatic protein, is also released by heparins from the endothelial cell surface. The absence of selectivity could restrict the utility of heparins as anti-cancer drugs. An additional major disadvantage of heparins is associated with their ability to induce bleeding, which limits the dosage to be prescribed for patients with malignancies.

Lately, a Cochrane analysis was published, evaluating the effectiveness and safety of heparins in ambulatory cancer patients. The study included 7,622 participants from 15 randomized controlled trials (RCTs) where either UFH or LMWH was employed. The meta-analysis showed an insignificant effect of the drugs on 12-month and 24-month mortality, with a decrease in VTE and an increase in minor bleeding. The authors concluded that the effect of heparins on the survival of cancer patients should be further assessed in relation to a specific type and stage of malignancy. Given the paucity of such data, at present, the judgment regarding the utility of heparin in a specific patient with cancer has to rely on an individual risk assessment, taking into account the patient’s quality of life.^18^

### Heparanase Procoagulant Activity Inhibition

Heparanase is an endoglycosidase able to cleave heparan sulfate (HS) side chains in restricted sites, causing liberation of HS fragments of approximately 5–7 kDa, which can still enhance anti-thrombin activity.^19^,^20^ Heparanase has been shown to augment tumor cell metastasis through HS cleavage and alteration of the extracellular matrix (ECM), increasing cell dissemination.^21^,^22^ Additionally, heparanase, released from activated cells of the immune system and promoting migration of vascular endothelial cells, appears to affect neovascularization, inflammation, and autoimmunity.^21^–^23^ Furthermore, high levels of heparanase expression have been documented in most human tumors, wound healing, and diabetic nephropathy.^21^–^23^ In contrast to multiple proteases that can degrade peptides in the ECM, heparanase is currently found to be the only human protein capable of degrading HS chains.^21^,^22^ Heparanase protein is mainly expressed in the placenta, platelets, granulocytes, and monocytes, with low or no expression in connective tissues and normal epithelia.^24^–^27^

We have previously demonstrated that heparanase regulates the expression of TF^28^ and releases TFPI from the endothelial cell surface membrane and tumor cells by direct interaction, intensifying cell surface coagulant activity.^29^ Additionally, we have shown that through a direct interaction with TF, heparanase actually serves as a cofactor in TF functioning, leading to elevated factor Xa production with ensuing coagulation system activation.^30^ Data supporting the procoagulant effect of heparanase have been published by Baker et al.,^31^ who have demonstrated in arterial injury and stent occlusion models that mice over-expressing heparanase produce a larger thrombus within a shorter time period compared to control animals.

While an elevated heparanase level in the plasma and biopsies of patients with cancer had been previously reported, we have recently explored the heparanase procoagulant activity in the plasma samples drawn at presentation from 65 patients with non-small cell lung cancer and compared the findings with those of 20 controls.^32^ Remarkably, not only was the heparanase antigen level higher in the patient group compared to controls (*P*=0.05), but also the heparanase procoagulant activity appears to be significantly increased in the patient cohort (*P*<0.0001). Furthermore, high heparanase procoagulant activity has been shown to correlate with a short survival (*P*=0.001). Therefore, high heparanase procoagulant activity could be a new mechanism of coagulation system activation in cancer, which might be further evaluated as a biomarker of patient survival.

The association of the coagulation system with cancer progression and inflammation has been further confirmed in our recent study where newly described peptides derived from TF pathway inhibitor 2 (TFPI-2) first Kunitz domain have demonstrated their ability to hinder the heparanase procoagulant activity by obstructing the interaction of TF with heparanase. Notably, *in vivo*, these peptides appear to significantly diminish activation of the coagulation system and sepsis severity induced by lipopolysaccharides, without putting the patient at risk of substantial bleeding.^33^ In another study, these peptides have significantly attenuated tumor growth and vascularization.^34^ Additionally, increased survival rate and a delay in tumor relapse have been demonstrated in mice treated with the peptides in question compared to control animals. Remarkably, neither in these two studies,^33^,^34^ nor in an inferior vena cava ligation model,^33^ was the side effect of bleeding detected. These results reinforce the evidence of existing interaction between cancer, inflammation, and the hemostatic system. Recently, Bochenek et al. have shown in a model of endothelial senescence that inhibition of heparanase activity using the TFPI-2 peptides prevents the enhanced venous thrombus formation in aged mice and restores it to the thrombotic phenotype of control adult mice.^35^ Thus, inhibition of heparanase may also potentially affect the process of aging.

## CONCLUSIONS

Optimizing the prevention of vascular events remains a challenge that could be tackled by better risk stratification and broader application of precision medicine. The primary focus of the present research is on the creation of heparin mimetics. Their limited effect on the coagulation system allows circumventing bleeding complications, while considerably obstructing the procoagulant activity of heparanase. Inhibition of heparanase may also be achieved with the above described peptides derived from TFPI-2. Their ability to attenuate sepsis, tumor growth, and endothelial cell aging makes them potential candidates for future translational and clinical research.

## Abbreviations

DIC: disseminated intravascular coagulation
HS: heparan sulfate
IL: interleukin
LMWH: low-molecular-weight heparin
NETs: neutrophil extracellular traps
PAI-1: plasminogen activator inhibitor
TF: tissue factor
TFPI: tissue factor pathway inhibitor
TFPI-2: TF pathway inhibitor 2
TNF: tumor necrosis factor
TTP: thrombotic thrombocytopenic purpura
UFH: unfractionated heparin
VTE: venous thromboembolism
vWF: von Willebrand factor
